# Serum Total Bilirubin With Hospital Survival in Adults During Extracorporeal Membrane Oxygenation

**DOI:** 10.3389/fmed.2022.914557

**Published:** 2022-06-24

**Authors:** Rui Huang, Min Shao, Cheng Zhang, Ming Fang, Mengmeng Jin, Xuan Han, Nian Liu

**Affiliations:** ^1^Department of Critical Care Medicine, The First Affiliated Hospital of Anhui Medical University, Hefei, China; ^2^Anhui Provincial Cancer Institute, The First Affiliated Hospital of Anhui Medical University, Hefei, China; ^3^Department of Respiratory and Critical Care, The Fourth Affiliated Hospital of Anhui Medical University, Hefei, China

**Keywords:** total bilirubin, hyperbilirubinemia, survival, extracorporeal membrane oxygenation, prognosis

## Abstract

**Background:**

Extracorporeal membrane oxygenation (ECMO) is widely used for refractory cardiopulmonary failure treatment. The disadvantage of ECMO is its higher risk profile and clinical resource consumption. This observation examines the role of serum total bilirubin (TBIL) as a predictor of adult patient outcomes on ECMO support.

**Methods:**

This retrospective observation reports a single-center experience with adults on ECMO support between 2018 and 2021. Data were collected regarding demographics, ECMO details, laboratory parameters, and outcomes. We examined the elevation of TBIL to predict survival and variables associated with hyperbilirubinemia.

**Results:**

The patients who died within 28 days had a twofold higher peak level of TBIL than those who survived [73.10 (38.60, 98.64) vs. 34.50 (24.03, 54.85); *P* = 0.003]. Univariate logistic regression analyses demonstrated that high TBIL was remarkably associated with an elevated risk of 28-day mortality (OR: 7.25; 95% CI: 2.31–25.49; *P* = 0.001) and total mortality (OR: 5.71; 95% CI: 1.82–20.66; *P* = 0.001). The TBIL value was 65 μmol/L as the best cut-off value, and the observation group was divided into a high TBIL subgroup (*n* = 21) or a low TBIL subgroup (*n* = 39). The demographic and clinical features did not show a difference, whereas Sequential Organ Failure Assessment (SOFA) and APACHE II scores and ALT, AST, and LAC before ECMO initiation correlated with high or low TBIL (*P* < 0.05). For coagulation function at the time of TBIL peak, the levels of prothrombin time (PT), activated partial thromboplastin time (APTT), prothrombin time activity (PTA), and fibrinogen (FIB) were different between the two subgroups (*P* < 0.05). The SOFA score was potentially associated with hyperbilirubinemia after ECMO initiation, and the prediction accuracy was 0.800.

**Conclusion:**

Serum total bilirubin elevation appears after ECMO initiation and correlates with survival, while other markers of liver injury do not. Serum total bilirubin is an easy-to-measure biomarker to be a predictor of survival after ECMO initiation.

## Background

Extracorporeal membrane oxygenation (ECMO) is increasingly used to treat refractory cardiopulmonary failure worldwide. Venovenous ECMO (VV-ECMO) is recommended for refractory hypoxia or hypoxic hypercapnia respiratory failure, whereas venoarterial ECMO (VA-ECMO) is recommended for refractory cardiac failure with/without respiratory failure (1, 2). ECMO should be used following the guidelines that have been demonstrated in ECMO centers to ensure its feasibility and safety (3). Understanding and knowledge of ECMO-related complications and predictors are part of the management protocols recommended by the Extracorporeal Life Support Organization (ELSO). A recent literature review (4) of 19 studies reported many predictors of ECMO-related complications, but the predictors were widely variable given the heterogeneity in patient severity, care, and training quality.

Bilirubin is a marker of liver dysfunction and has been included in a variety of scoring algorithms to assess critical patient prognosis (5, 6). Elevated serum bilirubin levels are not always induced by primary liver disease and may result from multiple conditions, such as hypoxia, toxins and drug injury, affecting liver function at different stages or simultaneously; therefore, elevated serum bilirubin is recognized as a marker of the generalized stress response (7, 8).

The relationship between liver function and survival has been investigated mainly in the context of VA-ECMO and postcardiac surgery (9), and few studies have reported on VV-ECMO (10), which may be related to systemic hypoperfusion. The Survival After Veno Arterial ECMO (SAVE) score defined liver injury before ECMO initiation. The serum bilirubin cut-off value was 33 μmol/L (1.93 mg/dl), and those of aspartate aminotransferase (AST) and alanine aminotransferase (ALT) were > 70 units/L (11). However, data on the fluctuation of these markers are not available, and cut-off values after the initiation of ECMO correlate with survival (12). In this study, we reported the association between elevated serum total bilirubin (TBIL) and adult patient outcomes during ECMO support, defined the cut-off value of TBIL and identified factors related to elevated TBIL. This information is a useful complement to ECMO management protocols and may help guide therapy when serum TBIL levels increase during ECMO support.

## Methods

**FIGURE 1:**
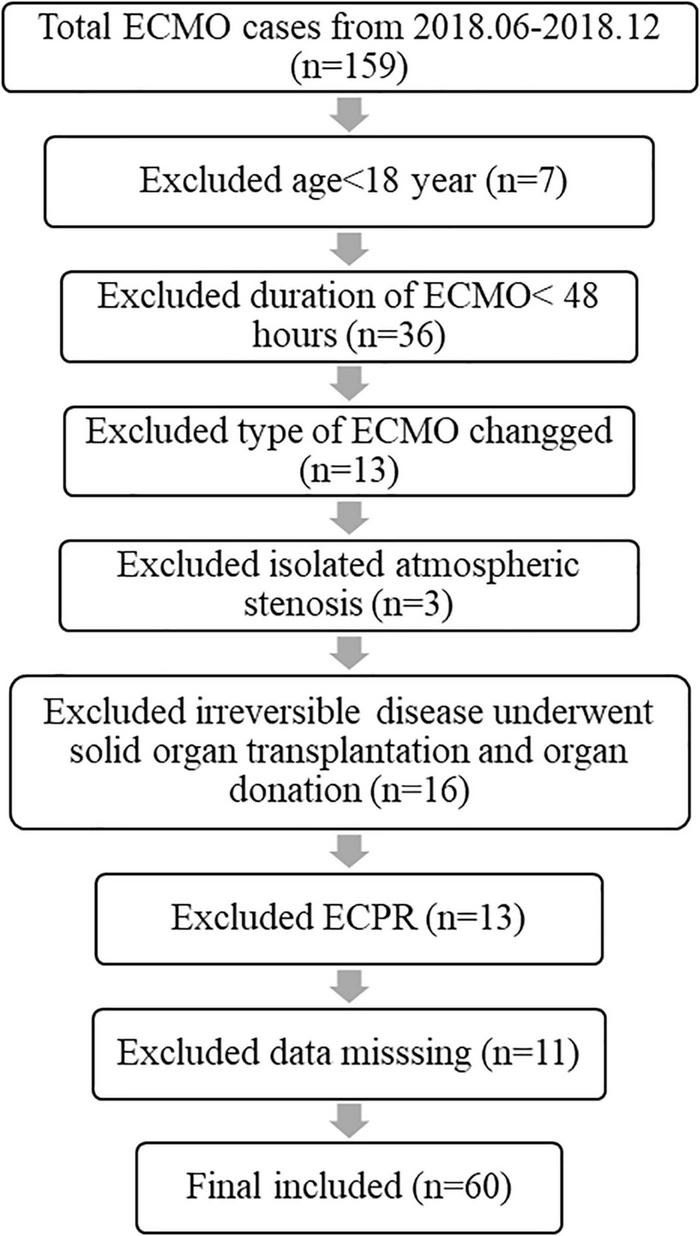
Flow diagram of patient enrolment. ECPR, extracorporeal cardiopulmonary resuscitation.

This retrospective study was approved by the Anhui Medical University Medical Institutional Review Board. All 159 patients during the past 3 years (2018–2021) were included in the retrospective study to objectively assess TBIL fluctuation and the relationship between TBIL levels and related survival during adult patient ECMO support. Many cases were excluded, and the exclusion criteria are presented in Figure 1. All ECMO patients’ uniform data, including demographic characteristics, details of ECMO, laboratory parameters, and outcomes, were reported through the Chinese Society of Extracorporeal Life Support Organization (CSELSO) registry and collected retrospectively.

The First Affiliated of Anhui Medical University, which offers all treatment modalities for acute and chronic cardiopulmonary failure, is a member of the CSELSO. All ECMO services were provided in the Critical Care Medicine Department, and the ECMO team was headed by the intensivists of our department. The systems used included Bio-Console 560 (Medtronic, Inc., Minneapolis, MN, United States) and Cardiohelp™ (Maquet Inc., Rastatt, Germany). ECMO team members received CSELSO certification to minimize practice differences through standardized care and were well trained. Intensive care unit (ICU) nurses on the ECMO team also received training similar to that of ECMO specialists to monitor the ECMO circulation system. Cannulation, circuit and pump management and other procedures were performed by an intensivist-led team in our department. IV heparin (20 units/kg) was administered 5–10 min before cannula implantation if there was no active bleeding or contraindications to anticoagulation. Unfractionated heparin was used for anticoagulation. Compared with Activated Clotting Time (ACT) assays, the activated partial thromboplastin time (APTT) better correlates with heparin concentrations during ECMO support (13). Heparin anticoagulant was monitored by APTT. We maintained the APTT at 50–70 s, and the ACT range was 180–200 s as previously described (14).

Relevant data about liver function, including lactate (LAC), albumin (ALB), TBIL, AST, ALT, alkaline phosphatase (ALP), hemoglobin (HB) and platelets (PLTs), were also collected. Values for each variable were collected before cannulation, and the highest and lowest values observed throughout the ECMO support process were recorded. According to the SAVE score, TBIL greater than 33 μmol/L was considered elevated (11) and defined as hyperbilirubinemia.

## Statistics

All statistical analyses were performed using RStudio (1.4.1717) and considered statistically significant when the *p*-value was less than 0.05. Categorized variables are described as numbers (percentages), and the chi-square test or Fisher’s exact test was used for comparison. The variable distribution was explored and visualized by histogram. Based on the distribution, continuous variables were reported as the mean (standard deviation) or median (interquartile range) and compared with Student’s *t*-test or Wilcoxon test between two independent groups. The correlation between two continuous variables is presented using dotted plots and calculated using the Spearman correlation coefficient. To obtain the odds ratio (OR), univariate and multivariate logistic regression analyses were performed. In multivariate analysis, continuous covariates were transformed into binary variables due to collinearity.

The optimal cut-off value for peak TBIL during ECMO was identified using 3 methods, i.e., receiver operating curve (ROC), Chi-square test, and logistic regression. For the ROC method, the optimal threshold should have the maximum sum of sensitivity and specificity. For the Chi-square test, the optimal threshold should have the greatest capacity to discriminate the two groups, i.e., the maximum value of the chi-square test statistic. For logistic regression, the optimal threshold should have the best univariate model fit, i.e., the lowest value of the Akaike information criterion (AIC) or the highest value of the concordance index (C-index).

Given that this study considered many serological and score parameters as covariates and collinearity frequently existed across these indicators, conventional logistic regression was inappropriate to explore the potential determinants for a given outcome. As a result, the random forest algorithm was used to incorporate all the possible descriptors without data transformation or engineering. This technique is a decision tree-based method that has no assumption about the data distribution or inner correlation. Both categorized and continuous data on different scales can be addressed. An importance value is given to a variable to indicate its contribution to the outcome. Variables with importance values greater than zero were further included in the prediction model, and the performance was evaluated by 10-fold cross validation. This process was implemented using the R package “mlr3verse.”

## Results

### Patient Enrolment and Baseline Characteristics

Cardiac failures were noted in 26 cases (43.3%), including 12 (20%) with acute myocardial infarction, 8 (13.3%) with acute fulminant myocarditis, 4 (6.7%) with stress cardiomyopathy, and 2 (3.3%) with acute pulmonary embolism. Respiratory failure was noted in 34 cases (56.7%), including 19 (31.7%) with severe pneumonia (bacteria or virus), 4 (6.7%) with acute interstitial pneumonia, 4 (6.7%) with aspiration, 2 (3.3%) with asthma, 2 (3.3%) with diffuse alveolar hemorrhage, and 3 (5%) with severe trauma. The baseline characteristics before ECMO initiation are described in Table 1. The patients who died within 28 days had a twofold higher peak level of TBIL than those who survived [73.10 (38.60, 98.64) vs. 34.50 (24.03, 54.85); *P* = 0.003]. Similar results were observed between the patients who eventually survived [67.38 (36.83, 98.56) vs. 30.86 (21.37, 50.65); *P* = 0.002]. The causes of death in 25 patients (41.6%) in this study included multiple organ failure (MOF) in 14 patients, septic shock in 7 patients, refractory cardiac failure in 2 patients, and bleeding in 2 patients.

**TABLE 1:** Baseline characteristics of the included patients stratified by 28-day mortality.

Variable	Survival (*N* = 35)	Death (*N* = 25)	*P*-value	Total (*N* = 60)
Sex (female)	14 (40.0)	11 (44.0)	0.965	25 (41.7)
Age	50.00 (35.00, 58.50)	59.00 (53.00, 69.00)	0.007	53.00 (40.75, 65.25)
BMI	23.63 (2.86)	22.62 (3.39)	0.217	23.21 (3.11)
IABP (present)	3 (8.6)	5 (20.0)	0.369	8 (13.3)
Tobacco smoking (yes)	7 (20.0)	5 (20.0)	> 0.999	12 (20.0)
Alcohol drinking (yes)	6 (17.1)	4 (16.0)	> 0.999	10 (16.7)
ECMO type (VV)	22 (62.9)	12 (48.0)	0.378	34 (56.7)
Limb ischemia (present)	1 (2.9)	2 (8.0)	0.764	3 (5.0)
Sepsis (present)	17 (48.6)	7 (28.0)	0.181	24 (40.0)
MV time (day)	11.00 (8.00, 30.00)	7.00 (5.00, 12.00)	0.006	10.00 (7.00, 19.75)
ECMO time (hour)	159.00 (117.50, 240.50)	146.00 (106.00, 193.00)	0.315	155.50 (110.75, 234.75)
Total ICU stay (day)	25.00 (12.00, 39.50)	9.00 (5.00, 13.00)	< 0.001	13.00 (9.00, 28.00)
Total hospital stay (day)	29.00 (16.50, 47.00)	13.00 (7.00, 22.00)	< 0.001	22.00 (12.00, 35.25)
Parameters before ECMO				
SOFA score	10.94 (3.05)	13.96 (2.62)	< 0.001	12.20 (3.22)
APACHE II score	27.00 (21.00, 30.00)	33.00 (32.00, 35.00)	< 0.001	29.50 (24.75, 33.00)
AST (U/L)	58.00 (35.55, 524.50)	217.20 (66.00, 887.00)	0.121	79.50 (39.75, 735.00)
ALT (U/L)	47.00 (28.50, 285.50)	72.00 (36.00, 402.00)	0.205	50.50 (29.00, 316.75)
TBIL (μmol/L)	16.00 (11.40, 24.20)	17.67 (11.30, 37.90)	0.264	16.14 (11.28, 26.97)
DBIL (μmol/L)	6.69 (4.20, 9.20)	9.18 (5.74, 33.92)	0.221	6.69 (4.40, 10.30)
ALB (g/L)	33.39 (6.03)	33.56 (5.56)	0.915	33.46 (5.79)
LAC (mmol/L)	2.66 (1.88, 3.34)	4.60 (3.20, 10.58)	< 0.001	3.10 (2.29, 5.97)
HB (g/L)	116.80 (27.90)	113.08 (27.47)	0.61	115.25 (27.55)
PLT (10^9/L)	194.63 (102.57)	168.36 (77.92)	0.286	183.68 (93.29)

*BMI, body mass index; IABP, intra-aortic balloon pumping; ECMO, Extracorporeal membrane oxygenation; ICU, intensive care unit; MV, mechanical ventilation; VV, Venovenous; APACHE, Acute Physiology and Chronic Health Evaluation; SOFA, Sequential Organ Failure Assessment; ALT, alanine transaminase; AST, aspartate aminotransferase; TBIL, total bilirubin; DBIL, direct bilirubin; ALB, albumin; LAC, lactate; HB, hemoglobin; PLT, platelet. Category variables were displayed with N (%); normally distributed variables were displayed with mean (standard deviation); skewed variables were displayed with median (P25, P75).*

### High Peak Total Bilirubin Levels Were Associated With the Risk of 28-Day and Total Mortality

**FIGURE 2:**
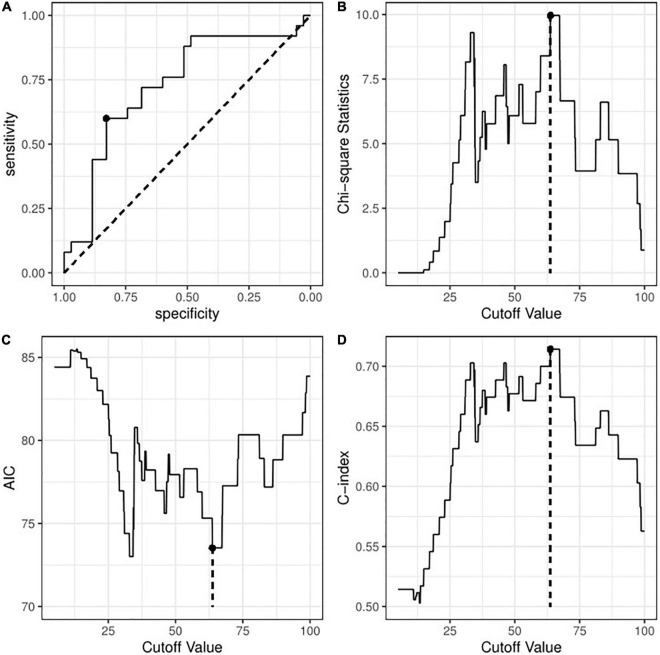
Identification of optimal cut-off value for peak TBIL based on **(A)** the receiver operating curve method; **(B)** Chi-square test with maximum statistics; **(C)** logistic regression with minimum AIC; or **(D)** logistic regression with maximum C-index. TBIL, total bilirubin; AIC, Akaike information criterion; C-index, concordance index.

We next transformed peak TBIL into a binary variable by determining the optimal cut-off value. Figure 2A shows that the threshold identified by the ROC method was 65.45 μmol/L with a sensitivity of 0.600 and a specificity of 0.829. Figures 2B–D consistently shows an optimal threshold ranging from 63.7 to 67.2 μmol/L. Taken together, we selected 65 μmol/L as the best cut-off value; thus, the patients were divided into a high TBIL subgroup (≥ 65 μmol/L; *N* = 21) or a low TBIL subgroup (< 65 μmol/L; *N* = 39). Table 2 describes the characteristics of the two subgroups. The demographic and clinical features did not differ between the two groups, whereas some parameters before ECMO initiation, including Sequential Organ Failure Assessment (SOFA), Acute Physiology and Chronic Health Evaluation II (APACHE II), ALT, AST and LAC, correlated with high or low TBIL (*P* < 0.05).

**TABLE 2:** Comparison of the characteristics between the patients with high and low TBIL during ECMO.

Variable	< 65 μ mol/L (*N* = 39)	≥ 65 μ mol/L (*N* = 21)	*P*-value	Total (*N* = 60)
Sex (female)	16 (41.0)	9 (42.9)	> 0.999	25 (41.7)
Age	51.00 (35.00, 63.50)	55.00 (51.00, 67.00)	0.127	53.00 (40.75, 65.25)
BMI	23.63 (2.80)	22.45 (3.56)	0.163	23.21 (3.11)
IABP (present)	4 (10.3)	4 (19.0)	0.577	8 (13.3)
Tobacco smoking (yes)	10 (25.6)	2 (9.5)	0.250	12 (20.0)
Alcohol drinking (yes)	8 (20.5)	2 (9.5)	0.468	10 (16.7)
ECMO type (VV)	23 (59.0)	11 (52.4)	0.827	34 (56.7)
Limb ischemia (present)	2 (5.1)	1 (4.8)	> 0.999	3 (5.0)
Sepsis (present)	16 (41.0)	8 (38.1)	> 0.999	24 (40.0)
Parameters before ECMO				
SOFA score	10.95 (2.90)	14.52 (2.44)	< 0.001	12.20 (3.22)
APACHE II score	28.00 (22.00, 32.00)	33.00 (31.00, 35.00)	0.001	29.50 (24.75, 33.00)
AST (U/L)	68.00 (35.55, 321.50)	308.00 (66.00, 1464.00)	0.019	79.50 (39.75, 735.00)
ALT (U/L)	46.00 (27.50, 82.00)	152.00 (38.00, 883.00)	0.025	50.50 (29.00, 316.75)
TBIL (μmol/L)	15.10 (11.25, 25.70)	21.50 (12.80, 29.80)	0.175	16.14 (11.28, 26.97)
DBIL (μmol/L)	6.00 (4.20, 9.20)	9.35 (7.32, 33.92)	0.051	6.69 (4.40, 10.30)
ALB (g/L)	34.47 (5.53)	31.58 (5.93)	0.065	33.46 (5.79)
LAC (mmol/L)	2.81 (2.15, 3.75)	4.60 (3.10, 9.92)	0.007	3.10 (2.29, 5.97)
HB (g/L)	119.82 (28.23)	106.76 (24.66)	0.080	115.25 (27.55)
PLT (10^9/L)	192.10 (86.86)	168.05 (104.61)	0.345	183.68 (93.29)

*BMI, body mass index; IABP, intra-aortic balloon pumping; ECMO, Extracorporeal membrane oxygenation; VV, Venovenous; SOFA, Sequential Organ Failure Assessment; ALT, alanine transaminase; AST, aspartate aminotransferase; TBIL, total bilirubin; DBIL, direct bilirubin; ALB, albumin; LAC, lactate; HB, hemoglobin; PLT, platelet. Category variables were displayed with N (%); normally distributed variables were displayed with mean (standard deviation); skewed variables were displayed with median (P25, P75).*

**FIGURE 3:**
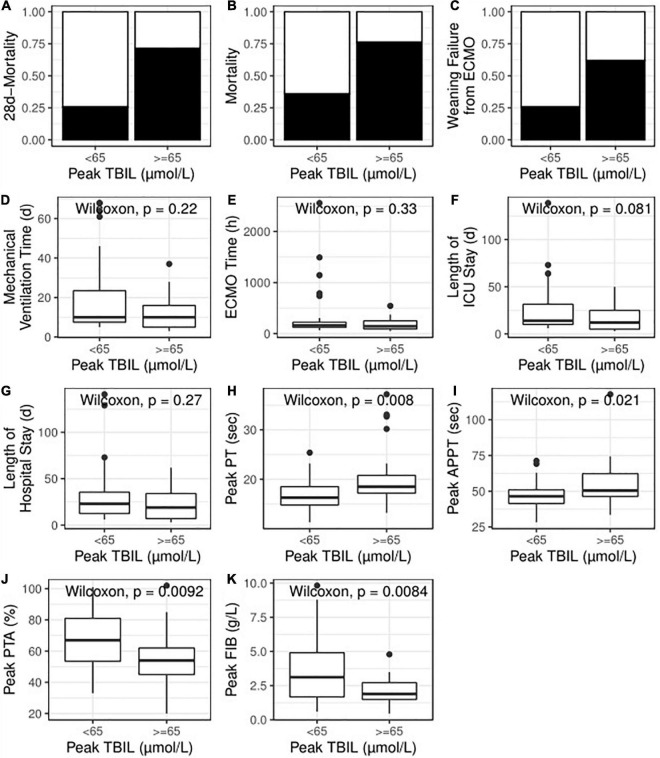
High TBIL during ECMO indicated poor primary outcome **(A,B)**, poor secondary outcome **(C–G)** and abnormal coagulation function **(H–K)**. TBIL, total bilirubin; ECMO, extracorporeal membrane oxygenation; ICU, intensive care unit; PT, prothrombin time, APTT, activated partial thromboplastin time; PTA, prothrombin time activity; FIB, fibrinogen.

Univariate logistic regression analyses demonstrated that high TBIL was remarkably associated with an elevated risk of 28-day mortality (OR: 7.25; 95% CI: 2.31–25.49; *P* = 0.001) and total mortality (OR: 5.71; 95% CI: 1.82–20.66; *P* = 0.001). Figures 3A,B also illustrates that the high TBIL subgroup had significantly more deceased cases (*P* < 0.05). After adjusting for age, ECMO type (VA vs. VV), sepsis (present vs. absent), LAC level before ECMO initiation (< 2 vs. ≥ 2) and APACHE II score before ECMO initiation (< 15 vs. ≥ 15), high TBIL was still correlated with the risk at 28 days (OR_adj_: 7.23; 95% CI: 1.98–31.72; *P*_adj_ = 0.004) and total mortality (OR_adj_: 4.79; 95% CI: 1.35–19.90; *P*_adj_ = 0.020).

### The Association Between Peak Total Bilirubin Level and Secondary Outcomes

**FIGURE 4:**
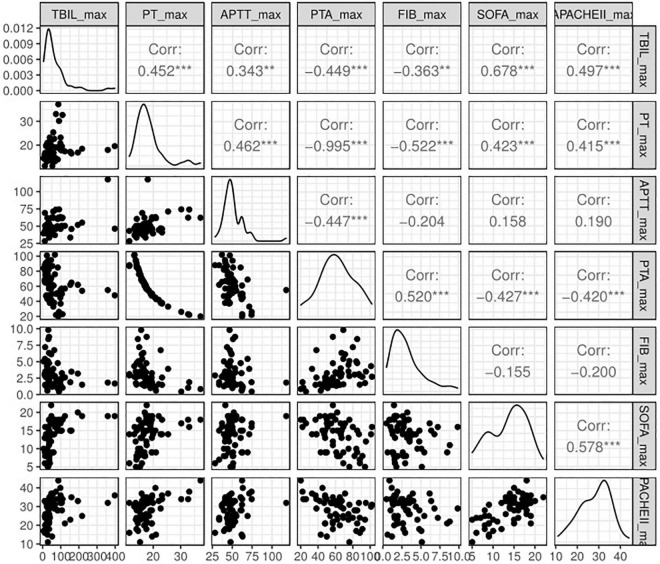
Spearman correlation analysis of peak TBIL and other simultaneous clinical parameters. TBIL, total bilirubin; PT, prothrombin time, APTT, activated partial thromboplastin time; PTA, prothrombin time activity; FIB, fibrinogen; SOFA, Sequential Organ Failure Assessment.

The patients successfully weaned from ECMO tended to have a lower peak level of TBIL [73.25 (37.62, 95.26) vs. 36.58 (24.00, 68.46)]; however, no statistical significance was observed (*P* = 0.225). Indeed, the high TBIL subgroup had a greater risk of failure weaning from ECMO according to univariate analysis (OR: 4.71; 95% CI: 1.55–15.36; *P* = 0.008; Figure 3C) and multivariate analysis (OR_adj_: 4.33; 95% CI: 1.33–15.32; *P*_adj_ = 0.018). For other secondary outcomes (i.e., duration of hospital stay, duration of ICU stay, mechanical ventilation time, and ECMO duration), no statistically significant differences were observed between the high and low TBIL subgroups (Figures 3D–G). For coagulation function at the time of peak TBIL, the prothrombin time (PT), APTT, prothrombin time activity (PTA), and fibrinogen (FIB) levels differed between the two subgroups (*P* < 0.05, Figures 3H–K). In addition, peak TBIL was significantly correlated with these parameters (Figure 4).

### Exploration of Potential Determinants That Could Predict the Occurrence of High Peak Total Bilirubin

**FIGURE 5:**
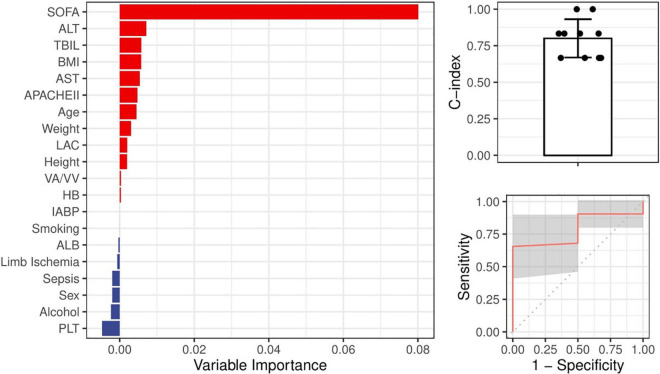
Ranking of the potential indictors for the occurrence of high TBIL during ECMO based on the random forest algorithm. All variables were demographic features or parameters obtained before ECMO. A greater importance value suggested a closer relationship between the variable and the occurrence of high TBIL during ECMO.

Given that a high peak level of TBIL was apparently associated with worse outcome, we investigated possible predictors of its occurrence. We incorporated all the demographic features, clinical features, and parameters before ECMO into a random forest algorithm to predict the occurrence of TBIL peak levels greater than 65 μmol/L. Figure 5 (left panel) demonstrates that 12 variables (red) were potentially associated with the outcome, of which the SOFA score before ECMO played the most important role. We next built a random forest prediction model with the 12 variables and obtained a prediction accuracy of 0.800 (Figure 5 right panel).

## Discussion

To date, studies assessing how liver injury before ECMO initiation correlates with survival have mainly focused on patients supported by VA-ECMO. A small cohort reported that the Model for End-stage Liver Disease excluding INR (MELD-XI) score pre-calculation had a strong association with increased mortality (15), and similar findings were reported the Model for End-Stage Liver Disease (MELD) score in 49 patients who underwent heart transplantation (16). A cohort reported 240 patients with postcardiotomy cardiogenic shock and found that ALP and TBIL before ECMO initiation were the strongest predictors of 30-day mortality (17). Recently, findings regarding the definition of liver dysfunction remain unclear, and unifying trends and prognostic markers are not uniform among reports in the literature. Notably, liver function may be more difficult to objectively quantify before ECMO initiation (18, 19), and in many observations, liver function before ECMO initiation is often normal (20). These studies generally show that liver injury strongly affects prognosis, which may be due to acquired hemostasis, refractory peripheral vasodilation shock caused by nitric oxide metabolism disorder, and the absence of immune capacity caused by progressive liver injury (21).

A retrospective institutional database query found that the relevant parameters of liver function are usually normal before VA-ECMO initiation, but abnormalities in various markers develop after ECMO initiation. Finally, both bilirubin and lactate elevations correlated with increased mortality (22). Using a quantitative model, we definitively demonstrate that hyperbilirubinemia after ECMO initiation is a dominant factor in decreased survival and establish 65 μmol/L as the best cut-off value.

AST and ALT values are widely recognized as indicators of liver injury given their rapid increase to an early peak. However, in this study, the two variables are not prognostic factors of survival. This finding is consistent with prior results (20). A multivariable model showed that AST and ALT failed to achieve statistical significance. This finding might be attributed to the notion that elevated ALT and AST levels are only related to acute liver injury. Of note, the patients who died within 28 days had a twofold higher peak TBIL levels than those who survived, and similar results were observed between the patients who eventually survived and those who did not. However, some parameters (i.e., SOFA, APACHE II, ALT, AST, and LAC) also increased with high TBIL (*P* < 0.05).

The frequency and severity of liver dysfunction prior to ECMO initiation in this study were not significantly different between survivors and non-survivors, and most patients had normal liver function before cannulation. Given that a peak value of TBIL > 65 μmol/L was associated with worse outcome, we assessed possible predictors of elevated TBIL using the random forest algorithm and found that 12 variables, including age, BMI, and lactate, were potentially associated with the outcome. Specifically, the SOFA score before ECMO played the most important role, yielding a prediction accuracy of 0.800. Persistently elevated bilirubin levels may indicate that the liver has not recovered even after the elimination of the initial liver injury or that other new complications occur, leading to liver injury. We also found that coagulation function, including PT, APTT, PTA, and FIB, was significantly correlated with the peak TBIL value, which is interpreted as acquired hemostatic abnormalities secondary to liver dysfunction. Thus, the knowledge that serum TBIL elevation exceeds certain cut-off values indicates decreasing survival and is beneficial for further decision-making.

## Limitations

The limitations of this study were that it was a single institution experience and a retrospective study. The primary diseases varied significantly, and we were unable to maintain homogeneity in therapeutic strategies, which may affect prognosis or add more residual confounders. The purpose of the study is to provide a reference for decision-making in patients on ECMO with hyperbilirubinemia, but the relatively low numbers of patients in this observation limited the statistical power.

Due to the relatively small sample size and heterogeneity of the participants, the confounders are unlikely to be fully addressed, especially for the undetected covariates. In real-world studies, instrument variables are able to balance the seen and unseen biases across the subgroups. Unfortunately, we failed to identify a possible instrumental variable from the available medical information, so we only adjusted the clinically and statistically important covariates that could be recognized. Therefore, the results could possibly extend current knowledge but should also be taken with caution due to the inherent limitations.

## Conclusion

The main results of the study were as follows: (a) the patients who died within 28 days had twofold higher peak TBIL levels than those who survived; (b) the best threshold identified was 65 μmol/L; (c) hyperbilirubinemia was correlated with the risk of 28-day and total mortality; (d) 12 variables were potentially associated with hyperbilirubinemia, and the SOFA score before ECMO played the most important role. In conclusion, during ECMO support, elevated total serum bilirubin levels appear to be consistently associated with survival, whereas other markers of liver injury are not associated with survival. Serum total bilirubin is an easy-to-measure biomarker that serves as a predictor of survival after ECMO initiation.

## Data Availability Statement

The raw data supporting the conclusions of this article will be made available by the authors, without undue reservation.

## Ethics Statement

The studies involving human participants were reviewed and approved by The First Affiliated Hospital of Anhui Medical University, the Committee on Medical Ethics. The patients/participants provided their written informed consent to participate in this study.

## Author Contributions

RH and NL designed and drafted the manuscript. RH, XH, MF, MS, and NL were involved in the clinical care and management of the patients. CZ and MJ analyzed the data. All authors approved the final manuscript as submitted and agreed to be accountable for all aspects of the work.

## Conflict of Interest

The authors declare that the research was conducted in the absence of any commercial or financial relationships that could be construed as a potential conflict of interest.

## Publisher’s Note

All claims expressed in this article are solely those of the authors and do not necessarily represent those of their affiliated organizations, or those of the publisher, the editors and the reviewers. Any product that may be evaluated in this article, or claim that may be made by its manufacturer, is not guaranteed or endorsed by the publisher.

## Abbreviations

ECMO: Extracorporeal membrane oxygenation
VV: Venovenous
VA: venoarterial
ELSO: Extracorporeal Life Support Organization
SAVE: Survival after venoarterial ECMO
AST: aspartate aminotransferase
ALT: alanine aminotransferase
TBIL: total bilirubin
DBIL: Direct bilirubin
CSELSO: Chinese Society of Extracorporeal Life Support Organization
LAC: lactate
ALB: albumin
ALP: alkaline phosphatase
HB: hemoglobin
PLTs: platelets
OR: odds ratio
ROC: receiver operating curve
AIC: Akaike information criterion
C-index: concordance index
BMI: Body mass index
IABP: Intra-aortic balloon pumping
ICU: Intensive care unit
MV: mechanical ventilation
SOFA: Sequential Organ Failure Assessment
APACHE: Acute Physiology and Chronic Health Evaluation
INR: International normalized ratio
MELD-XI: Model for End-stage Liver Disease excluding INR
MELD: Model for End-Stage Liver Disease
PT: prothrombin time
APTT: activated partial thromboplastin time
PTA: prothrombin time activity
FIB: fibrinogen
MOF: multiple organ failure.
